# Risk of complications after total hip arthroplasty in patients on testosterone replacement therapy

**DOI:** 10.1007/s00590-026-04774-2

**Published:** 2026-05-11

**Authors:** Farouk Khury, Sophia Antonioli, Garrett Ruff, Anzar Sarfraz, Eric Grossman, Joshua Rozell, Ran Schwarzkopf

**Affiliations:** 1 The Ruth and Bruce Rappaport Faculty of Medicine, Technion – Israel Institute of Technology, Haifa, Israel; 2 Department of Orthopedic Surgery, New York University Langone Orthopedic Hospital, New York, USA; 3 Division of Orthopedic Surgery, Rambam Health Care Campus, Haifa, Israel

**Keywords:** Testosterone replacement therapy (TRT), Total hip arthroplasty (THA), Periprosthetic joint infection (PJI), Dislocation

## Abstract

**Background:**

Testosterone replacement therapy (TRT) may cause side effects after orthopedic procedures. With total hip arthroplasty (THA) rates increasing, this study evaluates the relationship between TRT and postoperative complications in THA patients.

**Methods:**

A retrospective review in a large academic hospital was conducted of hypogonadal patients treated with TRT, who underwent primary, elective THA between 2012 and 2024. These were 1:2 propensity-matched based on age, body-mass index, and comorbidities to a “control” group that was not treated with TRT. Patient and TRT characteristics including serum testosterone levels, form of administration, 90-day emergency department visits (ED) and readmissions, reoperations and revisions were explored.

**Results:**

Among 152 patients aged 61.3 years who underwent THA with a 2.7-year follow-up, TRT was mainly administered intramuscularly (51.3%) or via transdermal gel (46.1%), followed by pellets (2.0%), and oral tablets (1.6%). Overall rates of 90-day ED visits and readmissions did not differ significantly between TRT and control patients (7.9% vs. 5.3%, *P* = 0.270 and 7.9% vs. 5.6%, *P* = 0.225, respectively). TRT patients had a significantly lower rate of 90-day ED visits due to surgery-related causes (0.7% vs. 2.3%, *P* = 0.048) but a significantly higher rate due to non-surgery-related causes (7.2% vs. 3.0%, *P* = 0.034). The incidence of PJI did not differ significantly between the groups (2.0% vs. 1.0%, *P* = 0.319). Reoperations and revisions were not different between the groups (*P* = 0.650 and *P* = 0.057, respectively). TRT administration form was not associated with 90-day ED visits (*P* = 0.380), readmissions (*P* = 0.563), reoperations (*P* = 0.441) or revisions (*P* = 0.669). Testosterone levels demonstrated a weak, negative, yet significant correlation with 90-day ED visits (*r* = -0.35, *P* = 0.040), but not with reoperations or revisions (*P* = 0.348 and *P* = 0.431, respectively).

**Conclusions:**

TRT in THA patients was associated with a reduced rate of surgery-related 90-day ED visits but an increased rate of non-surgery-related 90-day ED visits. Incidence of PJI and overall 90-day ED visits and readmission rates did not significantly differ. Administration form had no significant impact, while higher testosterone levels were linked to fewer 90-day ED visits. Although limited by its retrospective design and patient exclusions, further investigation is warranted to guide perioperative management in these patients, particularly given the known immunomodulatory effects of exogenous TRT.

## Introduction

While total hip arthroplasty (THA) is a well-tolerated and increasingly common procedure for managing hip osteoarthritis [1, 2], the target population demographics have become more diverse and complex, with higher rates of obesity, older age, and more comorbid conditions [3–5]. Among the comorbidities commonly seen in the arthritic population, the prevalence of adult-onset hypogonadism continues to rise. Studies estimate the prevalence of low serum testosterone levels is 10% in men in their 50s, 20% in their 60s, and 40% in their 70s [6, 7]. As a result, testosterone replacement therapy (TRT) prescriptions have increased threefold from 2001 to 2011, reaching 3.75% [8].

Hypogonadism, characterized by reduced serum testosterone levels, is associated with several pathologic states including reduced muscle strength and bone density [9], both of which are critical in the context of hip arthroplasty and postoperative rehabilitation. Although hypogonadism is commonly treated using TRT, treatment can be associated with increased rates of cardiovascular events after initiation [10] and immunosuppressive effects [11]. Furthermore, multiple studies have found higher rates of periprosthetic joint infections (PJI) in shoulder arthroplasty patients on TRT [12, 13]. Matsen et al. [13] reported a fourfold increase in PJI rates in this particular population, and Su et al. [12] suggested stopping TRT six months preoperatively to reduce rates of PJI. Notably, higher testosterone levels are linked to an increased presence of *Cutibacterium acnes* on the skin, as this is a common causative organism for shoulder PJI [14].

Although PJI with *Cutibacterium acnes* is less common after hip arthroplasty than in shoulder arthroplasty [15–17], PJI remains one of the most severe complications of THA. The significant morbidity, mortality, and healthcare costs due to PJI have been well described in previous literature [18, 19]. As the incidence of THA continues to increase [20], rates of subsequent PJI are also expected to rise [21], underscoring the importance of addressing modifiable risk factors like TRT to improve surgical outcomes. However, data on TRT’s effects on the THA population remain sparse. While a recent database analysis by Collins et al. [22] found that patients receiving TRT had higher revision and PJI rates following both hip and knee arthroplasties, this study lacked data on preoperative testosterone levels and detailed clinical characteristics, including causative PJI organisms.

Identifying and mitigating modifiable risk factors as well as understanding the impact of preoperative TRT are crucial to improving postoperative outcomes, particularly in the THA population, where data remains limited. Therefore, this study aims to (1) evaluate postoperative outcomes and complications in patients undergoing THA while receiving TRT, (2) assess whether the form of testosterone administration is associated with postoperative outcomes, and (3) examine the relationship between preoperative serum testosterone levels and the incidence of postoperative complications. We hypothesize that patients receiving TRT at the time of THA have higher rates of early postoperative complications compared to patients not receiving testosterone, and these outcomes may vary with serum testosterone levels but not with the form of administration.

## Methods

### Study design

A retrospective review was conducted of patients who underwent primary, elective THA between October 2012 and August 2024 at a single, urban, high-volume, academic hospital. Institutional Review Board approval was obtained prior to initiation of the study protocol. Patients were required to have a documented laboratory testosterone result or to have been prescribed any form of TRT within 30 days of undergoing surgery. TRT was identified through a review of electronic medical records (EMRs) for “testosterone” medication. Patients were included in the study if they were genetically male, older than 18 years of age, had undergone primary, elective THA indicated by either primary osteoarthritis, had complete electronic records and follow-up visits detailing the TRT as well as development of any postoperative complication, ED visit, readmission, reoperation or revision. Patients were excluded from the study if they had any diagnosis of ongoing or previous soft tissue, bone or joint infection, any ongoing or former immunosuppressant treatment, any active oncologic disease, or any incomplete data. The primary query yielded 1,125 patients. Following exclusion of 548 patients missing information of the form of TRT administered, 190 patients that were missing testosterone laboratory values, 166 patients who underwent revision arthroplasty, 58 patients who had a diagnosis other than primary osteoarthritis, and 11 lost to follow-up cases, the final study cohort consisted of 152 patients. A formal a priori power analysis was not performed, as this study retrospectively included all eligible patients who met the inclusion criteria. These TRT patients were propensity-score matched with a 1:2 ratio to a control cohort consisting of patients who underwent primary, elective THA and did not have any mention of “testosterone” in their EMR. The propensity-score matching was based on age, smoking status, body-mass index (BMI), American Society of Anesthesiologists score and Charlson Comorbidity Index (CCI).

### Data collection

Our institution’s EMR (Epic Caboodle. Version 15; Verona, Wisconsin, USA) was utilized to collect patients’ demographics (e.g., gender, age, BMI, race, smoking status, ASA class), past medical and surgical histories, form of TRT administration (transdermal gel or patch, intramuscular, pellets or oral tablets), past blood work and laboratory testosterone results, and past and ongoing TRT prescriptions. Quality metrics were also assessed, including 90-day emergency department (ED) visits—which were selected as an outcome measure to capture early postoperative healthcare utilization and acute medical complications, 90-day readmissions, reoperations, and revisions. Reoperations were defined as any operation excluding revision.

### Data analyses

Baseline patient characteristics were represented as counts with percentages for categorical variables and means ± standard deviations or ranges for continuous variables. In addition to descriptive statistics, the statistical model consisted of (1) *Chi*-squared (x^2^) test and Fisher’s exact test for assessing (1) correlation between the form of TRT administration and 90-day ED visit or readmission, reoperation, and revision, and (2) differences between TRT and control group in terms of 90-day ED visits and readmissions, reoperations and revisions, and (3) point-biserial analysis for evaluating the relationship between the most recent testosterone level and 90-day ED visit or readmission, reoperation, and revision. The propensity scores were estimated using logistic regression, and matching was performed using the Nearest Neighbour method. A balance test was performed to assess the quality of the match, which resulted in standardized mean differences (SMD) of 0.035 for the age match, 0.114 for the smoking status match, 0.035 for the BMI match, and 0.083 for the CCI match. This match was considered successful as all SMD values were below 0.2. Significance was defined as a *P*-value less than 0.05. In cases where a significant finding was observed, effect size (ES) was calculated for Cohen’s d (parameters: 0.20–0.50 small, 0.50–0.80 medium, > 0.80 large), and Cramer’s V (parameters: 0.10–0.30 small, 0.30–0.50 medium, > 0.50 large). All statistical analyses were conducted using SPSS Statistics (Version 28; IBM Corporation, Armonk, New York, USA).

### Baseline characteristics

The 152 TRT patients were aged 61.3 years (range, 35 to 85 years) with a BMI of 31.3 kg/m^2^ (range, 19.4 to 49.2) and CCI of 2.8 ± 2.8. The majority of the TRT and control patients were White (79.6% vs. 79.3%, *P* = 0.088), former smokers (40.1% vs. 53.3%, *P* = 0.494) with an ASA score III (52.6% vs. 55.6%, *P* = 0.889). Mean length of follow-up was 2.7 and 2.6 years in the TRT and control group, respectively, with no significant difference (*P* = 0.690). Mean length of stay was not significantly different between the groups (38.6 vs. 40.6 h, *P* = 0.568) (Table 1).

**Table 1:** Baseline patient characteristics comparing TRT and control group

Patient characteristic	TRT (*n* = 152)	Control (*n* = 304)	*P*-value (ES)
Mean age at surgery [range], (years)	61.3 [35–85]	61.7 [26–90]	0.725^t^
Mean BMI, (kg/m^2^)	31.3 [19.4–49.2]	31.5 [17.7–47.8]	0.715^t^
Race, n (%) White Black or African American Other	121 (79.6) 14 (9.2) 17 (11.2)	241 (79.3) 29 (9.2) 34 (11.2)	0.088^c^
Smoking status, n (%) Never Former Current	52 (34.2) 61 (40.1) 39 (25.7)	126 (41.4) 162 (53.3) 16 (5.3)	0.494^c^
ASA score, n (%) I II III IV	7 (4.6) 62 (40.8) 80 (52.6) 3 (2.0)	16 (5.3) 112 (36.8) 169 (55.6) 7 (2.3)	0.889^f^
Mean CCI ± SD	2.8 ± 2.8	2.9 ± 2.5	0.377^t^
Mean length of follow-up ± SD, (years)	2.7 ± 2.7	2.6 ± 2.5	0.690^t^
Mean length of stay [range], (hours)	38.6 [6-245]	40.6 [7-291]	0.568^t^

n, Number; %, Percentage; TRT, testosterone replacement therapy; SD, Standard deviation; BMI, Body-mass index; ASA, American Society of Anesthesiologists; CCI, Charlson Comorbidity Index; ^c^, *Chi*-squared test; ^F^, Fisher’s exact test; ^t^, Independent-sample *t*-test; ES, Effect size; *, Significance set at *P* < 0.05

TRT was mainly administered either intramuscularly (*n* = 78, 51.3%) or using a transdermal gel or cream (*n* = 70, 46.1%), with only select cases of pellet (*n* = 3, 1.9%) and oral tablet (*n* = 1, 0.7%) use. The most recent testosterone value prior to the THA was 279.3 ± 344.1 ng/dL (Table 2).

**Table 2:** Testosterone parameters among patients in the TRT group

Parameter	TRT (*n* = 152)
Mean testosterone value ± SD (ng/dL) (normal range: 300–1,000)	279.3 ± 344.1
*Form of TRT administration, n (%)*	
Topical gel or cream	70 (46.1)
Intramuscular	78 (51.3)
Pellets	3 (1.9)
Oral tablets	1 (0.7)

n, Number; %, Percentage; TRT, testosterone replacement therapy; SD, Standard deviation

## Results

### Outcomes and complications

The differences in outcomes and complications between the groups are shown in Table 3. Within 90 days following THA, there were no significant differences in ED visits between TRT and control patients (7.9% vs. 5.3%, *P* = 0.270). Further stratification demonstrated significantly lower rate of 90-day ED visits due to surgery-related causes in TRT patients compared to controls (0.7% vs. 2.3%, *P* = 0.048). The effect size (ES) of this observation was medium (Cramer’s V = 0.388). The surgery-related causes from 90-day ED visit were pain in the operated hip joint in the TRT group (*n* = 1, 0.7%), periprosthetic fracture (*n* = 2, 0.7%), pain in the operated hip joint (*n* = 2, 0.7%), periprosthetic joint infection (PJI) (*n* = 1, 0.3%), superficial surgical site infection (SSSI) (*n* = 1, 0.3%), and dislocation (*n* = 1, 0.3%) in the control group. TRT patients had a significantly higher rate of non-surgery-related 90-day ED visits compared to the control patients (7.2% vs. 3.0%, *P* = 0.034). The ES of this finding was negligible (Cramer’s V = 0.098), as it fell slightly below the predefined threshold for a small effect. Among the non-surgery-related causes for 90-day ED visits, TRT patients had a significantly higher rate of cardiovascular, gastrointestinal, and urogenital events compared to the control group (5.9% vs. 2.3%, *P* = 0.047). Other causes included venous thromboembolism/pulmonary embolism (VTE/PE) (*n* = 1, 0.7%), and cellulitis (*n* = 1, 0.7%) in the TRT group, and cellulitis (*n* = 2, 0.7%) in the control group (Table 3).

**Table 3:** Comparison of postoperative outcomes and complications between TRT and control group

Outcome	TRT (*n* = 152)	Control (*n* = 304)	*P*-value (ES)
**90-day ED visit**, n (%) Surgery-related cause PJI SSSI Periprosthetic fracture Dislocation Pain Non-surgery-related cause VTE/PE Cellulitis Other (cardiovascular, gastrointestinal, urogenital)	12 (7.9) 1 (0.7) 0 (0.0) 0 (0.0) 0 (0.0) 0 (0.0) 1 (0.7) 11 (7.2) 1 (0.7) 1 (0.7) 9 (5.9)	16 (5.3) 7 (2.3) 1 (0.3) 1 (0.3) 2 (0.7) 1 (0.3) 2 (0.7) 9 (3.0) 0 (0.0) 2 (0.7) 7 (2.3)	0.270^c^ 0.048*^F^ **(0.388)** 0.667^**F**^ 0.667^**F**^ 0.444^**F**^ 0.667^F^ 0.705^F^ 0.034*^c^ **(0.098)** 0.333^F^ 0.705^F^ 0.047*^c^ **(0.093)**
**90-day readmission**, n (%) Surgery-related cause PJI SSSI Periprosthetic fracture Dislocation Pain Non-surgery-related cause VTE/PE Cellulitis Other (cardiovascular, gastrointestinal, urogenital)	12 (7.9) 4 (2.6) 2 (1.3) 0 (0.0) 0 (0.0) 0 (0.0) 2 (1.3) 8 (5.3) 1 (0.7) 1 (0.7) 6 (3.9)	16 (5.3) 10 (3.3) 4 (1.3) 1 (0.3) 1 (0.3) 3 (1.0) 1 (0.3) 6 (2.0) 0 (0.0) 1 (0.3) 5 (1.6)	0.225^c^ 0.177^c^ 0.650^F^ 0.667^F^ 0.667^F^ 0.444^F^ 0.259^F^ 0.214^c^ 0.333^F^ 0.999^c^ 0.192^F^
**Reoperation**, n (%) Debridement due to SSSI DAIR due to PJI (*S. aureus*)	2 (1.3) 0 (0.0) 2 (1.3)	4 (1.3) 1 (0.3) 3 (1.0)	0.650^F^ 0.667^F^ 0.540^F^
**Revision**, n (%) PJI (*S. epidermidis)* Dislocation Aseptic loosening	1 (0.7) 1 (0.7) 0 (0.0) 0 (0.0)	6 (2.0) 0 (0.0) 3 (1.0) 3 (1.0)	0.057^F^ 0.333^F^ 0.295^F^ 0.295^F^
**Time to revision** [range] (days)	114	562 [89 − 1,808]	N/A
**Incidence of PJI**, n (%) *S. epidermidis* *S. agalactiae*	3 (2.0) 3 (2.0) 0 (0.0)	3 (1.0) 2 (0.7) 1 (0.3)	0.319^F^ 0.209^F^ 0.667^F^

n, Number; %, Percentage; TRT, Testosterone replacement therapy; ED, Emergency department; PJI, Periprosthetic joint infection; SSSI, Superficial surgical site infection; DAIR, Debridement, antibiotic, irrigation and implant retention; VTE, Venous thromboembolism; PE, Pulmonary embolism; *S. aureus*, *Staphylococcus aureus*; *S. epidermidis*, *Staphylococcus epidermidis*; *S. agalactiae*, *Streptococcus agalactiae*; ^c^, *Chi*-squared test; ^F^, Fisher’s exact test; ^t^, Independent-sample *t*-test; ES, effect size; N/A, Not applicable; *, Significance set at *P* < 0.05

There were no significant differences in 90-day readmission rates in the TRT group compared to controls (7.9% vs. 5.3%, *P* = 0.225), neither due to surgery-related causes (2.6% vs. 3.3%, *P* = 0.177), nor due to non-surgery-related causes (5.3% vs. 2.0%, *P* = 0.214). Surgical-related causes for 90-day readmissions were PJI (*n* = 2, 1.3%) and pain in the operated hip joint (*n* = 2, 1.3%) in the TRT group, and PJI (*n* = 4, 1.3%), dislocation (*n* = 3, 1.0%), SSSI (*n* = 1, 0.3%), periprosthetic fracture (*n* = 1, 0.3%), and pain (*n* = 1, 0.3%) in the control group. Non-surgery-related causes for 90-day readmissions were either cardiovascular, gastrointestinal, or urogenital (*n* = 6, 3.9%), venous thromboembolism or pulmonary embolism (VTE/PE) (*n* = 1, 0.7%), and cellulitis (*n* = 1, 0.7%) in the TRT group, and cardiovascular, gastrointestinal, or urogenital (*n* = 5, 1.6%) and cellulitis (*n* = 1, 0.3%) in the control group.

In terms of reoperations, two patients (1.3%) in the TRT cohort required debridement, irrigation, antibiotic and implant retention (DAIR), and four patients (1.3%) in the control group required DAIR (*n* = 3, 1.0%) and debridement (*n* = 1, 0.3%) following THA. This reoperation rate was not significantly different between the groups (*P* = 0.650). In terms of revisions, one patient (0.7%) in the TRT group underwent a two-stage revision arthroplasty 114 days after the primary THA, not significantly different (*P* = 0.057) from the six patients (2.0%) in the control group requiring revision due to dislocation (*n* = 3, 1.0%) and aseptic loosening (*n* = 3, 1.0%) 89 to 1,808 days following primary THA. TRT patients required revision earlier than control patients (114 vs. 562 days). The incidence of PJI necessitating an operative intervention was not significantly different in the TRT group compared to control patients (2.0 vs. 1.0%, *P* = 0.319). TRT patients had positive *Staphylococcus epidermidis* cultures (*n* = 3, 2.0%), while control patients had both *Staphylococcus epidermidis* (*n* = 2, 0.7%) and *Streptococcus agalactiae* (*n* = 1, 0.3%) cultures.

### Effect of TRT form

*Chi-*square test of independence was performed to assess the association between the categorical variables, and did not demonstrate any significant association between 90-day ED visit (*P* = 0.380) or readmission (*P* = 0.563), reoperation (*P* = 0.441), and revision (*P* = 0.669) and the TRT form of administration.

### Effect of testosterone levels

Correlations between testosterone levels and outcomes were determined using point-biserial analysis. This test demonstrated a weak negative, yet significant correlation between the testosterone level and 90-day ED visits (*r*=-0.35, *P* = 0.040). However, no significant findings were observed with either reoperation or revision rate (*P* = 0.348 and 0.431, respectively).

## Discussion

This study reveals a complex relationship between TRT and early postoperative outcomes following THA. We found a negatively weak, yet significant correlation between preoperative serum testosterone levels and 90-day ED visits (*P* = 0.040), suggesting that higher testosterone levels were associated with a reduced risk of early hospital utilization. While overall 90-day ED visit and readmission rates did not significantly differ between the TRT and control groups, analysis of specific causes provided more granular insight. TRT patients had a significantly lower rate of 90-day ED visits due to surgery-related causes (*P* = 0.048), but a significantly higher rate due to non-surgery-related causes (*P* = 0.034). Importantly, the incidence of PJI did not differ significantly between the groups. Similarly, the rates of reoperation and revision also showed no significant differences. The most common surgery-related complications observed were PJIs, dislocations, and aseptic loosening. Given the rarity of these complications [23] and the sample size of 152 TRT patients, the lack of a significant overall difference in these events is not unexpected. Furthermore, the form of TRT administration did not significantly impact any postoperative outcome.

Previous research reported an increased risk of PJI in both hip and knee arthroplasty patients on TRT when compared to matched controls [22]. Additionally, more 90-day medical complications and longer length of stay were reported in the TRT group [22]. These findings of higher non-surgery-related complications (e.g., cardiovascular, gastrointestinal, or urogenital events) in the TRT group are partially aligned with previous research by Collins et al. [22] who also reported more 90-day medical complications in TRT patients undergoing total joint arthroplasty. While that study found significantly higher rates of reoperations and revisions in the TRT group compared to control patients (3.6% vs. 2.3% odds ratio (OR) 1.46 with 95% confidence interval (CI) 1.21–1.75), our analysis showed no such significant difference. This difference may be due to the varied patient cohorts or study methodologies, though our cohort did have a numerically higher incidence of PJI (2.0% vs. 1.0%, *P* = 0.319).

Although there is limited additional research surrounding the PJI risk in THA patients on TRT, the literature surrounding PJI risk after shoulder arthroplasty supports this overarching theme that supplemental testosterone may increase infection risk after arthroplasty procedures. It has been reported that TRT increases the risk of PJI after shoulder arthroplasty up to 1.44-fold [12, 13, 16]. This heightened risk is thought to be related to the increased presence of *Cutibacterium acnes* on the skin in men with higher testosterone levels, as *C. acnes* is a common causative organism for shoulder PJI [12, 15, 16]. While certain risk factors have correlated to increased risk of PJI while on TRT [24, 25], Su et al. [12] documented that the timing of TRT may affect the incidence of PJI, particularly within six months of shoulder arthroplasty. Although our study did not investigate the time intervals of TRT and rate of PJI, future studies ought to explore this possible association.

Coupled with previous research [22], our finding of a negative correlation between serum testosterone level and 90-day ED visits (*r*=-0.35, *P* = 0.040) highlights an interesting association. However, correlation does not imply causation; it is highly possible that higher serum testosterone levels in this cohort simply reflect a better overall health status, nutritional baseline, or medication compliance rather than exerting a direct protective effect against early hospital utilization. It has been previously proposed that exogenous TRT may have an immunomodulatory effect [26–28]. Furman et al. [26] found that men with elevated testosterone levels exhibited lower antibody responses to an influenza vaccine than comparable cohorts. Furthermore, TRT has shown benefit in patients with autoimmune disease, most specifically systemic lupus erythematosus (SLE), as the androgens act to decrease the hyperactive immune response and decrease the frequency of SLE flares [27, 28]. If elevated testosterone levels have been linked to a weakened immune response, this could contribute to TJA patients on TRT having an increased risk of postoperative infections. These hypotheses should be further investigated.

The pharmacokinetics of testosterone can vary significantly based on the route by which the therapy is administered. For instance, transdermal applications such as testosterone creams and patches allow for more stable serum testosterone levels compared to intramuscular injections, which can lead to peaks and troughs in hormone levels [29, 30]. Subcutaneous testosterone can maintain stable serum testosterone concentrations similar to those achieved by IM injections [31]. Our study found that the form of TRT administration did not significantly impact postoperative outcomes. This suggests that the method of testosterone delivery, whether intramuscular injections, transdermal gels, or other forms, may not influence the risk of complications following THA. Further research is warranted to explore this topic.

### Limitations

Our study has several limitations, including the retrospective design and inherent potential for selection bias as well as incomplete data. Furthermore, as a single-center retrospective study, our review may not capture all postoperative events, such as ED visits, readmissions, reoperations, or revisions that occurred at outside hospital systems. Additionally, we acknowledge that in terms of identifying postoperative incidents, 90-day ED visits may be influenced by factors unrelated to surgical complications, such as comorbidities, or healthcare access. Our analysis was limited in regard to finding a correlation between testosterone level and postoperative complications since some patients did not have a recorded serum testosterone level. Furthermore, while patients were required to have a documented laboratory testosterone result or to have been prescribed any form of TRT within 30 days of undergoing surgery, the retrospective nature of this study limited our ability to precisely ascertain the total duration of TRT exposure prior to surgery, as well as patient adherence to or continuation of TRT postoperatively. These factors may significantly influence the observed outcomes and should be investigated in future prospective studies. Moreover, while we employed propensity-score matching to account for confounding variables, unmeasured factors, such as operative time or hemoglobin A1c level, may have influenced the observed outcomes, as these factors have been correlated to increase the risk of PJI [14]. Furthermore, our study is limited by the small sample size for certain methods of testosterone administration. This prevents us from fully assessing whether subcutaneous or intramuscular delivery, which may provide more stable testosterone levels, could affect immune response and alter infection risk [14]. Additionally, a large number of patients were excluded (548 patients) due to lacking sufficient data regarding their specific form of TRT administration in their medical charts, alongside 190 patients missing laboratory testosterone values. While a preliminary analysis of the excluded patients found no significant differences in baseline demographics compared to the included cohort, this substantial exclusion rate still introduces the possibility of an unmeasured selection bias. Consequently, the findings of this study should be interpreted with caution, as the final cohort may not perfectly represent the broader population of all patients receiving TRT. Lastly, a larger cohort could better evaluate rates of rare outcomes such as PJI, dislocation, and revisions, further contributing to our understanding of how TRT impacts these events.

## Conclusion

In conclusion, our study suggests that the use of TRT in patients undergoing THA is associated with a reduced rate of surgery-related 90-day ED visits but an increased rate of non-surgery-related 90-day ED visits. Overall 90-day ED visit and readmission rates did not significantly differ between the TRT and control groups. The incidence of PJI and rates of reoperation and revision did not significantly differ between the two groups. Furthermore, the form of TRT administration had no significant impact on any postoperative outcome, while higher serum testosterone levels were linked to fewer 90-day ED visits. However, these findings must be interpreted with caution due to the study’s retrospective design, substantial patient exclusion rate, and limitations in fully characterizing the total duration of preoperative testosterone exposure and postoperative adherence. Nonetheless, these findings underscore the importance of careful perioperative management and risk mitigation for non-surgical complications in patients on TRT that are undergoing THA. Despite the known immunomodulatory effects of exogenous TRT, further investigation is warranted to guide the perioperative management of hypogonadal patients on testosterone supplementation undergoing THA.

## Data Availability

No datasets were generated or analysed during the current study.
